# Association between activated partial thromboplastin time and septic shock in pregnant women: a retrospective cohort study

**DOI:** 10.3389/fmed.2026.1747286

**Published:** 2026-01-20

**Authors:** Shengjia Zheng, Dunjin Chen, Yuanmei Gao

**Affiliations:** 1 Department of Respiratory and Critical Care Medicine, Guangdong Provincial Key Laboratory of Major Obstetric Diseases, Guangdong Provincial Clinical Research Center for Obstetrics and Gynecology, The Third Affiliated Hospital of Guangzhou Medical University, Guangzhou, China; 2 Department of Obstetrics and Gynecology, Guangdong Provincial Key Laboratory of Major Obstetric Diseases, Guangdong Provincial Clinical Research Center for Obstetrics and Gynecology, Guangdong-Hong Kong-Macao Greater Bay Area Higher Education Joint Laboratory of Maternal-Fetal Medicine, The Third Affiliated Hospital of Guangzhou Medical University, Guangzhou, China

**Keywords:** activated partial thromboplastin time, coagulopathy, maternal mortality, pregnancy, sepsis, septic shock

## Abstract

Maternal sepsis is a life-threatening infectious complication, with septic shock linked to high mortality. Coagulopathy is a key feature, but the association between activated partial thromboplastin time (APTT) and maternal septic shock remains unclear. This retrospective study included 1,631 pregnant women with infection admitted from January 2017 to March 2023. Based on admission APTT levels, patients were divided into three groups: <28 s (*n* = 697), 28–42 s (*n* = 915), and >42 s (*n* = 19). A U-shaped relationship was observed between APTT and septic shock risk. Compared to the reference group (APTT 28–42 s), the group with APTT >42 s had a higher incidence of septic shock (57.9% vs. 11.8%), with a multivariable-adjusted odds ratio (OR) of 3.85 (95% CI, 0.94–15.78, *p* = 0.061). APTT <28 s was also associated with increased risk (adjusted OR 1.5; 95% CI, 1.02–2.2; *p* = 0.04). Threshold analysis identified 27.564 s as a critical point. In addition, each 1-s increase in APTT raised shock risk by 8.7%. Monitoring APTT may facilitate risk assessment in obstetric sepsis.

## 1 Introduction

Maternal sepsis, a life-threatening condition triggered by severe infection, may arise at any stage from pregnancy through childbirth to the post-abortion or postpartum period, typically manifesting as organ dysfunction (1). Its fatality rate disproportionately exceeds that of other pregnancy-related complications. Critically, maternal sepsis elevates neonatal mortality risk and contributes to persistent maternal morbidity, including chronic pelvic pain, tubal occlusion, and infertility (2). Responsible for 11% of all maternal deaths, it substantially amplifies mortality attributed to other causes (3). As the primary driver of Intensive Care Unit (ICU) fatalities in this population, maternal sepsis remains a major global contributor to maternal morbidity and mortality (4). Progression to septic shock is a key concern: sepsis in pregnant women can rapidly escalate to systemic infection and shock following diagnosis (5). Although septic shock complicates only 0.002–0.01% of deliveries and pregnant individuals represent only 0.3–0.6% of sepsis patients (6–8), its clinical impact is severe. Afessa et al.’s study of 74 obstetric ICU patients reported incidences of 59% for systemic inflammatory response syndrome (SIRS), 24% for severe sepsis, and 3% for septic shock (9). Notably, septic shock in pregnancy-compared to severe sepsis-correlates with significantly higher rates of disseminated intravascular coagulation (DIC), altered mental status, hyperbilirubinemia, and profound multiorgan failure (10). Early intervention, timely recognition, delivery management, and complication control is vital for reducing mortality.

Coagulopathy’s central role in sepsis pathogenesis is well-established. This systemic disturbance stems from synergistic mechanisms: platelet activation, coagulation cascade initiation, dysregulated inflammation or immunity, mitochondrial injury, neuroendocrine imbalance, and endoplasmic reticulum stress, collectively exacerbating multiorgan failure (11). Within this pathological framework, sepsis-associated coagulopathy is a critical complication with high mortality. Sepsis-induced coagulopathy (SIC) often progresses to microthrombus formation, DIC, and ultimately organ failure (12). The underlying mechanism involves systemic inflammatory response and immune activation triggered by pathogens, driving excessive release of inflammatory cytokines, such as interleukin-6 (IL-6) and tumor necrosis factor-alpha (TNF-*α*) (13–15). These mediators promote widespread inflammation while simultaneously activating the coagulation system. Endothelial injury caused by microbial infection further amplifies coagulation by increasing tissue factor exposure and impairing anticoagulant pathways (11). This series of pathological changes ultimately leads to SIC characterized by systemic microthrombi, and the subsequent consumption of coagulation factors along with disruption of hemostatic balance can result in typical DIC.

The unique physiological state of pregnant individuals exacerbates the aforementioned risks. Physiological adaptations, immunological changes, and anatomical alterations during pregnancy increase susceptibility to infection, particularly in the postpartum period, and may accelerate disease progression. Among these, changes in the hemostatic system are especially critical: hemostatic function gradually increases throughout gestation, peaking at term and creating a physiological hypercoagulable state (16, 17). When sepsis occurs, the frequency of thrombocytopenia increases significantly, and its severity independently predicts poor outcomes, including higher 28-day mortality, bleeding risk, and organ dysfunction (18–20). In recent years, several emerging biomarkers such as soluble platelet regulators and the tissue plasminogen activator-plasminogen activator inhibitor-1 complex (tPA/PAI-1 complex) have shown potential for early warning of septic shock and sepsis-induced DIC (21). In contrast, activated partial thromboplastin time (APTT), one of the key indicators in routine coagulation monitoring, has not been sufficiently explored regarding its significance in maternal sepsis, particularly in the development of septic shock. Existing limited evidence suggests that prolonged APTT may independently predict the occurrence of sepsis and septic shock, warranting special attention in the pregnant population. This study investigates whether APTT is associated with septic shock occurrence in pregnant and postpartum women.

## 2 Materials and methods

### 2.1 Patient selection

**Figure 1:**
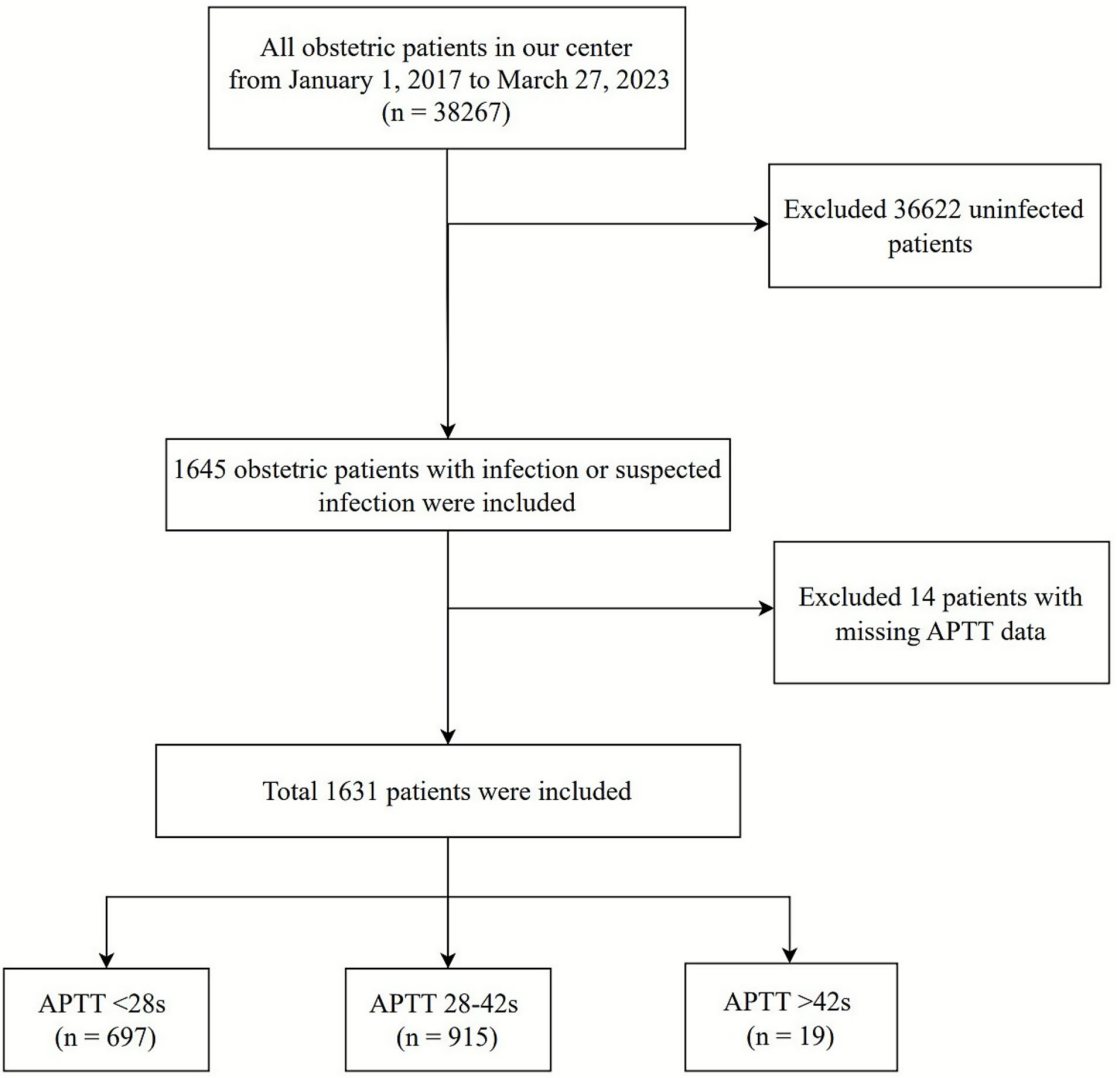
Flow chart of patient selection.

This retrospective cohort analysis involved 38,267 obstetric patients admitted to The Third Affiliated Hospital of Guangzhou Medical University from 1 January 2017 to 27 March 2023. After excluding 36,622 non-infected patients, 1,645 cases with suspected or confirmed infection were identified. Fourteen patients lacking APTT measurements were further excluded, resulting in a final cohort of 1,631 subjects (Figure 1). Participants were stratified by APTT intervals: <28 s (*n* = 697), 28–42 s (*n* = 915), and >42 s (*n* = 19). The primary outcome was septic shock occurrence. Ethical approval was obtained from the institutional review board, and written informed consent was secured from all participants prior to enrolment. All procedures complied with relevant guidelines and regulations, including the Declaration of Helsinki.

### 2.2 Diagnostic criteria

Sepsis is defined as life-threatening organ dysfunction resulting from a dysregulated host response to infection (22). Diagnosis was based on Sepsis-3 criteria, requiring both suspected or confirmed infection and an acute increase in the Sequential Organ Failure Assessment (SOFA) score of ≥2 points (13). Septic shock, a more severe subset of sepsis associated with greater mortality risk, involves circulatory failure and cellular or metabolic abnormalities. It is specifically defined as sepsis requiring vasoactive agents to maintain a mean arterial pressure ≥65 mmHg and exhibiting a serum lactate level >2 mmol/L despite adequate fluid resuscitation (13). Postpartum hemorrhage (PPH) was defined as blood loss ≥500 mL within 24 h of vaginal delivery or ≥1,000 mL following cesarean section (23).

### 2.3 Collection of clinical data

Medical records provided comprehensive maternal demographic data (age, body mass index (BMI), gravidity, parity), clinical characteristics (gestational age at admission, pregnancy or postpartum status, comorbidities including hypertension, diabetes, anemia, urinary tract infection, and thyroid diseases), and critical care parameters. These included PPH incidence, life-support interventions [vasoactive agents, mechanical ventilation, Extracorporeal Membrane Oxygenation (ECMO), continuous veno-venous hemofiltration (CVVH)], organ dysfunction scores [Glasgow Coma Scale (GCS), Acute Physiology and Chronic Health Evaluation II (APACHE II) and SOFA], and laboratory values (complete blood count, hepatic and renal function, coagulation markers: prothrombin time (PT); international normalized ratio (INR); APTT; fibrinogen; calcium; lactate) from the first laboratory tests after admission. Patient and fetal outcomes were assessed for septic shock development, ICU admission, and mortality. The primary outcome, septic shock during hospitalization, was compared across these APTT strata to determine prognostic significance.

### 2.4 Statistical analysis

Continuous variables are presented as mean ± standard deviation (SD) for normally distributed data or median (interquartile range [IQR]) for non-normally distributed data. Categorical variables are expressed as frequencies (percentages). Differences between the three predefined APTT cohorts (<28 s, 28–42 s, >42 s) were evaluated using one-way ANOVA for normally distributed continuous variables, Kruskal-Wallis tests for non-normally distributed continuous variables, and Pearson’s χ^2^ or Fisher’s exact tests for categorical variables. The association between APTT levels and maternal septic shock occurrence was assessed using multivariable logistic regression, with APTT modeled categorically by tertiles (<28 s, 28–42 s, >42 s) and the middle tertile (28–42 s) designated as the reference group. To systematically address potential confounding, we fitted three progressively adjusted logistic regression models. Model 1 controlled for demographic and basic obstetric factors maternal age, BMI, gravidity, parity, gestational weeks, and pregnancy stage. Model 2 additionally adjusted for comorbidities hypertension, diabetes, anemia, urinary tract infection, and thyroid diseases. Model 3 further incorporated laboratory parameters White Blood Cell Count (WBC), platelet count, Alanine Aminotransferase (ALT), and creatinine. Subgroup analyses examined the consistency of the APTT-septic shock association across strata of age, BMI, gestational weeks, parity, ICU admission status, and pregnancy stage. To elucidate the dose–response relationship, restricted cubic splines (RCS) modeled potential non-linear associations between continuous APTT values and septic shock risk. A two-piecewise logistic regression model was subsequently employed to formally identify and characterize any threshold effect suggested by the RCS analysis. All statistical analyses were conducted using R software version 3.3.2 (http://www.R-project.org, The R Foundation) and Free Statistics software version 2.2.1. Missing continuous variables were imputed using covariate means, while missing categorical variables were assigned specific codes (99 or 999) to retain cases in the analytical dataset.

## 3 Results

### 3.1 Baseline characteristics of participants

**Figure 2:**
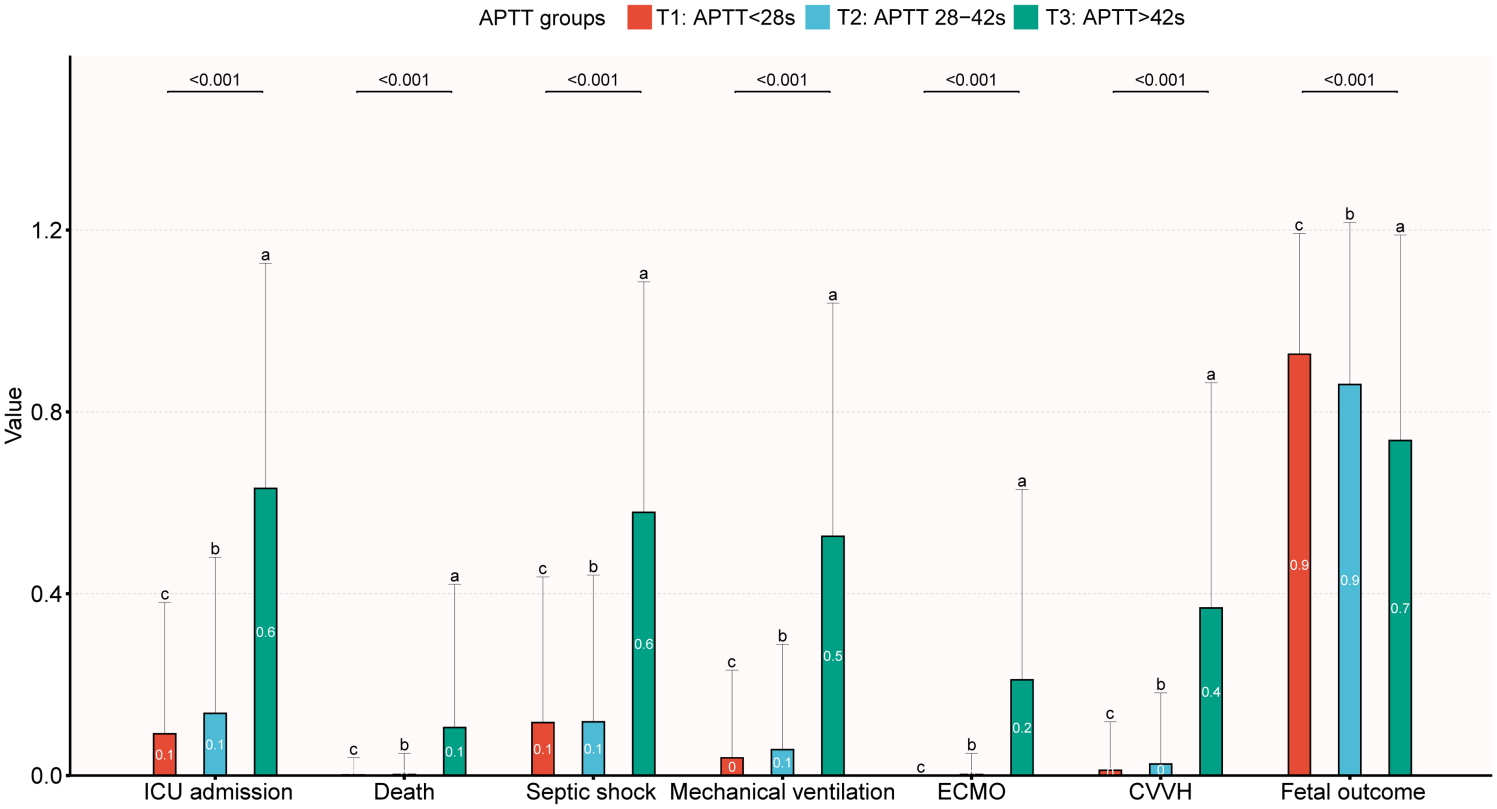
Primary and secondary outcomes across APTT groups. The letters a, b, and c are used to show statistically significant differences between variables. For all variables with the same letter, the difference between the means is not statistically significant. If two variables have different letters, they are significantly different. ICU, intensive care unit; ECMO, extracorporeal membrane oxygenation; CVVH, continuous veno-venous hemofiltration.

This retrospective cohort study analyzed 1,631 pregnant women categorized by APTT to assess clinical outcomes. Participants were stratified into three groups: <28 s (T1 group, *n* = 697), 28–42 s (T2 group, *n* = 915), and >42 s (T3 group, *n* = 19). Baseline characteristics and outcomes are summarized in Table 1 and Figure 2. The mean age across all groups was 31.2 ± 5.1 years, with no significant intergroup differences (*p* = 0.091).

**Table 1:** Characteristics of populations.

Variables	Total (*n* = 1,631)	APTT groups			*p*-value
		T1, APTT<28 s (*n* = 697)	T2, APTT 28-42S (*n* = 915)	T3, APTT>42 s (*n* = 19)	
Age, Mean ± SD	31.2 ± 5.1	31.5 ± 4.8	31.0 ± 5.3	30.1 ± 6.1	0.091
BMI, Mean ± SD	25.4 ± 4.2	25.9 ± 4.2	25.1 ± 4.1	24.9 ± 4.5	0.004
Gravidity, Mean ± SD	2.4 ± 1.5	2.4 ± 1.5	2.3 ± 1.5	2.6 ± 1.4	0.256
Parity, *n* (%)					0.355
Unipara	722 (44.6)	315 (45.3)	403 (44.4)	4 (22.2)	
Multipara	898(55.4)	380 (54.7)	504(55.6)	14 (77.8)	
Gestational week, Mean ± SD	31.3 ± 9.4	32.2 ± 8.1	30.7 ± 10.1	29.3 ± 13.6	0.004
Stage, *n* (%)					< 0.001
Antenatal period	1,440 (88.3)	655 (94)	774 (84.6)	11 (57.9)	
Postpartum period	191 (11.7)	42 (6)	141 (15.4)	8 (42.1)	
Hypertension, *n* (%)					0.182
No	1,477 (90.6)	641 (92)	818 (89.4)	18 (94.7)	
Yes	154 (9.4)	56 (8)	97 (10.6)	1 (5.3)	
Diabetes, *n* (%)					0.72
No	1,333 (81.7)	565 (81.1)	753 (82.3)	15 (78.9)	
Yes	298 (18.3)	132 (18.9)	162 (17.7)	4 (21.1)	
Anemia, *n* (%)					0.078
No	1,012 (62.0)	421 (60.4)	583 (63.7)	8 (42.1)	
Yes	619 (38.0)	276 (39.6)	332 (36.3)	11 (57.9)	
Urinary tract infection, *n* (%)					0.772
No	1,371 (84.1)	591 (84.8)	764 (83.5)	16 (84.2)	
Yes	260 (15.9)	106 (15.2)	151 (16.5)	3 (15.8)	
Thyroid diseases, *n* (%)					0.986
No	1,504 (92.2)	642 (92.1)	844 (92.2)	18 (94.7)	
Yes	127 (7.8)	55 (7.9)	71 (7.8)	1 (5.3)	
PPH, *n* (%)					0.094
No	1,458 (89.4)	624 (89.5)	820 (89.6)	14 (73.7)	
Yes	173 (10.6)	73 (10.5)	95 (10.4)	5 (26.3)	
Vasoactive drugs, *n* (%)					< 0.001
No	1,554 (95.3)	673 (96.6)	871 (95.2)	10 (52.6)	
Yes	77 (4.7)	24 (3.4)	44 (4.8)	9 (47.4)	
Mechanical ventilation, *n* (%)					< 0.001
No	1,542 (94.5)	670 (96.1)	863 (94.3)	9 (47.4)	
Yes	89 (5.5)	27 (3.9)	52 (5.7)	10 (52.6)	
ECMO, *n* (%)					< 0.001
No	1,625 (99.6)	697 (100)	913 (99.8)	15 (78.9)	
Yes	6 (0.4)	0 (0)	2 (0.2)	4 (21.1)	
CVVH, *n* (%)					< 0.001
No	1,593 (97.7)	689 (98.9)	892 (97.5)	12 (63.2)	
Yes	38 (2.3)	8 (1.1)	23 (2.5)	7 (36.8)	
GCS, Mean ± SD	14.6 ± 1.7	14.8 ± 1.1	14.6 ± 1.9	10.2 ± 5.6	< 0.001
SOFA, Median (IQR)	0.0 (0.0, 1.0)	0.0 (0.0, 0.0)	0.0 (0.0, 1.0)	7.0 (1.5, 8.5)	< 0.001
APACHE II, Mean ± SD	16.6 ± 7.2	14.2 ± 6.6	16.9 ± 7.0	23.9 ± 7.6	< 0.001
WBC,10^9/L, Mean ± SD	10.8 ± 5.1	10.8 ± 4.2	10.7 ± 5.6	10.2 ± 8.1	0.858
RBC,10^12/L, Mean ± SD	3.9 ± 1.3	3.8 ± 1.7	3.9 ± 1.0	3.5 ± 1.1	0.193
PLT, 10^9/L, Mean ± SD	227.1 ± 87.7	232.1 ± 77.6	224.8 ± 93.6	155.5 ± 113.1	< 0.001
PCT, ng/mL, Median (IQR)	0.1 (0.1, 0.3)	0.1 (0.1, 0.2)	0.1 (0.1, 0.4)	2.1 (1.1, 16.7)	< 0.001
AST, U/L, Median (IQR)	16.1 (12.7, 22.0)	15.6 (12.5, 19.2)	16.9 (13.0, 24.0)	32.4 (23.9, 58.6)	< 0.001
ALT, U/L, Median (IQR)	10.8 (7.9, 16.9)	9.9 (7.5, 14.6)	11.5 (8.1, 18.1)	50.9 (9.3, 176.3)	< 0.001
Tbil, umol/L, Median (IQR)	5.0 (3.6, 7.7)	4.7 (3.4, 6.8)	5.2 (3.7, 8.1)	14.3 (7.0, 28.0)	< 0.001
ALB, g/L, Mean ± SD	32.0 ± 5.4	32.4 ± 4.7	31.8 ± 5.9	28.8 ± 5.9	0.003
BUN, mmol/L, Median (IQR)	3.0 (2.3, 4.0)	3.0 (2.3, 3.9)	3.0 (2.3, 4.0)	3.5 (2.9, 6.0)	0.029
Cr, umol/L, Median (IQR)	51.0 (44.0, 60.0)	50.0 (43.0, 58.0)	52.0 (45.0, 61.0)	81.0 (55.5, 128.5)	< 0.001
Ca^2+^, mmol/L, Mean ± SD	2.1 ± 0.2	2.1 ± 0.2	2.1 ± 0.2	2.7 ± 0.9	< 0.001
Lactate, mmo/LMean ± SD	1.6 ± 0.8	1.7 ± 0.8	1.6 ± 0.8	1.6 ± 1.1	0.209
PT, Mean ± SD	11.0 ± 1.5	10.6 ± 0.9	11.1 ± 1.5	16.7 ± 4.9	< 0.001
INR, Mean ± SD	1.0 ± 0.1	1.0 ± 0.1	1.0 ± 0.1	1.5 ± 0.4	< 0.001
APTT, Mean ± SD	29.2 ± 5.2	26.0 ± 1.5	31.0 ± 2.8	58.0 ± 22.4	< 0.001
Fbg, g/L, Mean ± SD	4.3 ± 1.3	4.3 ± 0.9	4.3 ± 1.2	4.4 ± 8.0	0.66
MAP, Mean ± SD	88.7 ± 12.9	88.2 ± 11.3	89.1 ± 13.7	84.7 ± 26.6	0.134
Septic shock, *n* (%)					< 0.001
No	1,431 (87.7)	616 (88.4)	807 (88.2)	8 (42.1)	
Yes	200 (12.3)	81 (11.6)	108 (11.8)	11 (57.9)	
ICU admission, *n* (%)					< 0.001
No	1,430 (87.7)	633 (90.8)	790 (86.3)	7 (36.8)	
Yes	201 (12.3)	64 (9.2)	125 (13.7)	12 (63.2)	
Death, *n* (%)					< 0.001
No	1,626 (99.7)	696 (99.9)	913 (99.8)	17 (89.5)	
Yes	5 (0.3)	1 (0.1)	2 (0.2)	2 (10.5)	
Fetal outcome, *n* (%)					< 0.001
Death	188 (11.5)	52 (7.5)	131 (14.3)	5 (26.3)	
Survival	1,439 (88.2)	644 (92.4)	781 (85.4)	14 (73.7)	

BMI, body mass index; PPH, postpartum hemorrhage; ECMO, Extracorporeal Membrane Oxygenation; CVVH, Continuous Veno-Venous Hemofiltration; GCS, Glasgow Coma Scale; SOFA, Sequential Organ Failure Assessment; APACHE II, Acute Physiology and Chronic Health Evaluation; WBC, white cell count; Hb, Hemoglobin; RBC, red blood cell count; PLT, platelet; PCT, procalcitonin; ALB, Albumin; ALT, Alanine transaminase; AST, Aspartate transaminase; Tbil, Total bilirubin; Cr, creatinine; BUN, Blood Urea Nitrogen; Fbg, Fibrinogen; INR, Prothrombin international ratio; PT, prothrombin time; APTT, activated partial thromboplastin time; ICU, intensive care unit; MAP, mean arterial pressure.

Compared to T2 group, the T1 group showed superior outcomes, including a lower postpartum hemorrhage rate (6.0% vs. 15.4%) and reduced need for critical interventions such as vasoactive drugs (3.4% vs. 4.8%), mechanical ventilation (3.9% vs. 5.7%), ECMO (0% vs. 0.2%), and CVVH (1.1% vs. 2.5%; all *p* < 0.001 unless specified). Severity scores further supported this advantage: T1 group had lower APACHE II (14.2 ± 6.6 vs. 16.9 ± 7.0) scores, alongside higher GCS (14.8 ± 1.1 vs. 14.6 ± 1.9; all *p* < 0.001). Laboratory parameters indicated better coagulation function in T1 group, with reduced PT (10.6 ± 0.9 s vs. 11.1 ± 1.5 s), and higher platelet counts (232.1 ± 77.6 vs. 224.8 ± 93.6 × 10^9^/L; *p* < 0.001). Hepatic and renal function markers were also more favorable.

Conversely, the T3 group displayed significantly worse outcomes than T2 group. Utilization of life-supporting therapies increased substantially, including vasoactive drugs (47.4% vs. 4.8%), mechanical ventilation (52.6% vs. 5.7%), ECMO (21.1% vs. 0.2%), and CVVH (36.8% vs. 2.5%; all *p* < 0.001). Severity scores confirmed this deterioration: SOFA [7.0 (1.5–8.5) vs. 0.0 (0.0–1.0)], APACHE II (23.9 ± 7.6 vs. 16.9 ± 7.0), and lower GCS (10.2 ± 5.6 vs. 14.6 ± 1.9; all *p* < 0.001). T3 group also exhibited severe coagulopathy, characterized by prolonged PT (16.7 ± 4.9 s vs. 11.1 ± 1.5 s), elevated INR (1.5 ± 0.4 vs. 1.0 ± 0.1), and thrombocytopenia (155.5 ± 113.1 vs. 224.8 ± 93.6 × 10^9^/L; *p* < 0.001). Multi-organ dysfunction was evident through abnormal liver enzymes, renal impairment, and hypoalbuminemia (all *p* < 0.05).

Clinically, T3 group had higher rates of septic shock (57.9% vs. 11.8%, *p* < 0.001) and ICU admission (63.2% vs. 13.7%, *p* < 0.001). Fetal mortality was also significantly elevated in T3 group (26.3% vs. 14.3%, *p* < 0.001). These findings demonstrate that APTT exceeding 42 s serves as a critical indicator of multiorgan dysfunction and adverse outcomes in obstetric sepsis, necessitating urgent clinical intervention.

In addition, the overall maternal mortality rate in the cohort was 0.3% (5/1631). Fetal mortality occurred in 11.5% (188/1631) of cases. As detailed in Table 1, both maternal and fetal mortality rates varied significantly across APTT groups (both *p* < 0.001). The mortality rate was highest in the T3 group (APTT >42 s), with 10.5% (2/19) maternal mortality and 26.3% (5/19) fetal mortality.

### 3.2 Association between APTT levels and maternal septic shock

**Figure 3:**
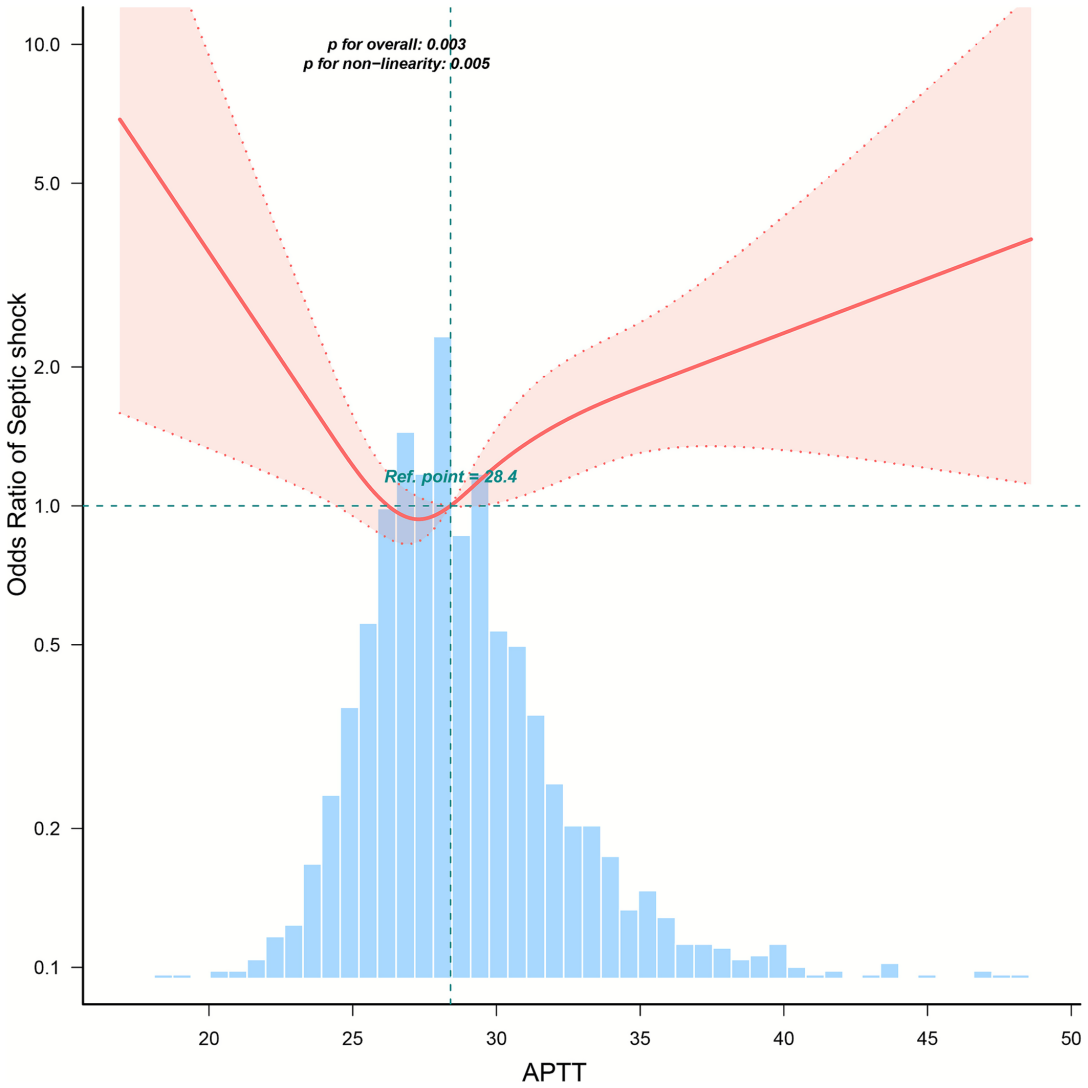
Restricted cubic spline analysis of the relationship between APTT and septic shock risk.

In this cohort of 1,631 pregnant women, multivariable regression analysis revealed a non-linear relationship between APTT levels and the risk of septic shock (Figure 3, *p* < 0.05). As shown in Table 2, using the T2 group (APTT 28–42 s) as the reference, patients in the T3 group (APTT > 42 s) had significantly higher odds of septic shock in the unadjusted model (OR 10.27, 95% CI 4.04–26.11; *p* < 0.001). This association remained statistically significant after adjusting for clinical variables in Model 1 (OR 5.83, 95% CI 1.63–20.82; *p* = 0.007) and Model 2 (OR 5.57, 95% CI 1.58–19.65; *p* = 0.008), and showed a trend toward significance in the fully adjusted Model 3 (OR 3.85, 95% CI 0.94–15.78; *p* = 0.061). Conversely, patients in the T1 group (APTT < 28 s) showed an increased risk in the fully adjusted model (OR 1.5, 95% CI 1.02–2.2; *p* = 0.04), although this was not significant in the crude or partially adjusted models. Furthermore, threshold analysis identified an inflection point at 27.564 s (Table 3), indicating that each 1-s increase beyond 27.6 s was associated with an 8.7% increase in risk (OR 1.087, 95% CI 1.03–1.15; *p* = 0.0015).

**Table 2:** Multivariable logistic regression analysis of the association between APTT tertiles and septic shock.

	Crude Model^a^		Model 1^b^		Model 2^c^		Model 3^d^	
Variable	OR (95% CI)	*p*-value	OR (95% CI)	*p*-value	OR (95% CI)	*p*-value	OR (95% CI)	*p*-value
APTT groups								
T2 (APTT 28–42 s)	1 (Ref)		1 (Ref)		1 (Ref)		1 (Ref)	
T1 (APTT<28 s)	0.98 (0.72–1.34)	0.91	1.45 (1.00–2.10)	0.05	1.41 (0.97–2.05)	0.073	1.5 (1.02–2.20)	0.04
T3 (APTT>42 s)	10.27 (4.04–26.11)	<0.001	5.83 (1.63–20.82)	0.007	5.57 (1.58–19.65)	0.008	3.85 (0.94–15.78)	0.061

^a^Crude model: No adjusted.

^b^Model 1: Adjusted for age, BMI, gravidity, parity, gestational week and pregnancy stage.

^c^Model 2: Adjusted for Model 1 plus hypertension, diabetes, anemia, urinary tract infection and thyroid disease.

^d^Model 3: Adjusted for Model 2 plus WBC, PLT, ALT and Cr.

**Figure 4:**
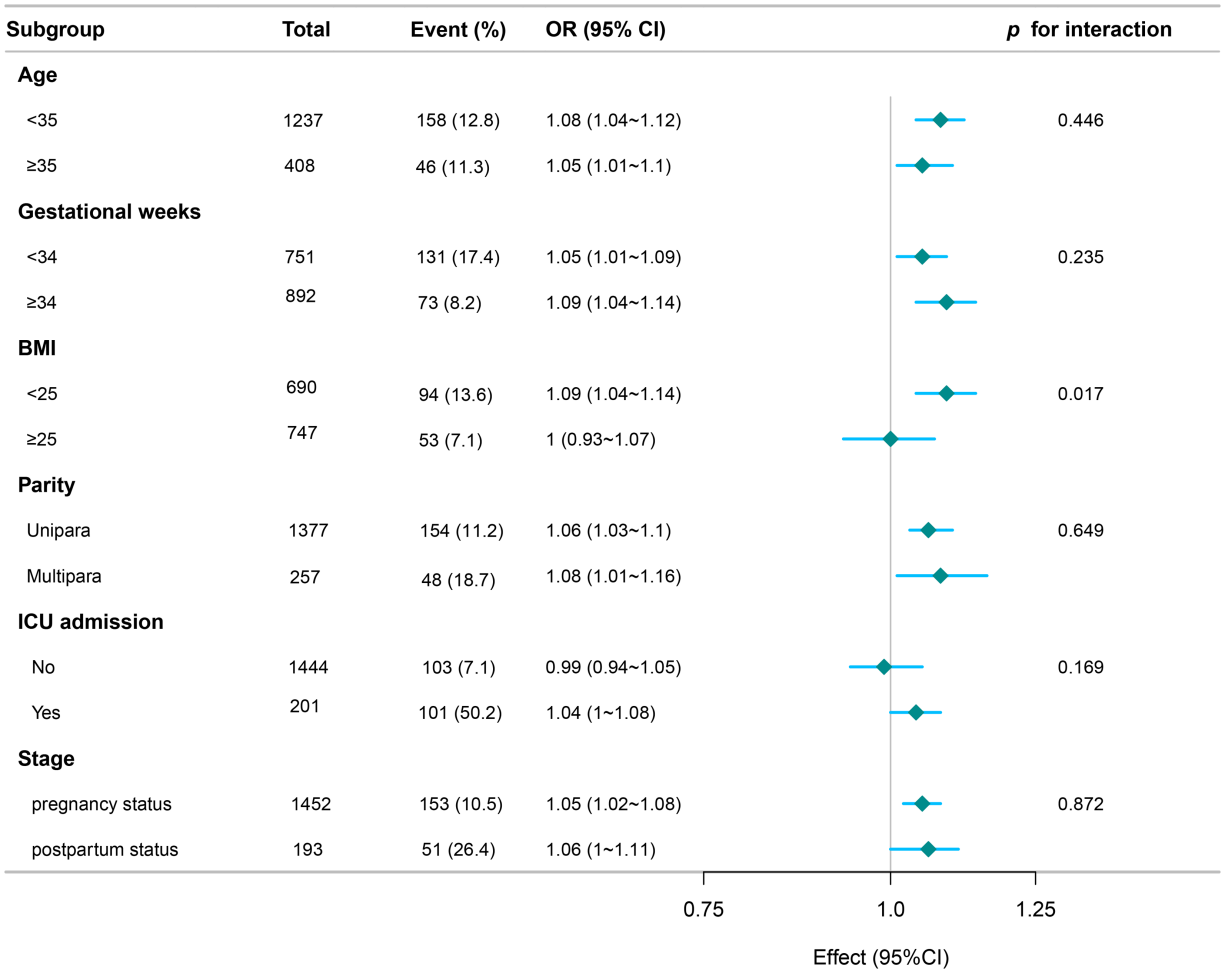
Forest plot of subgroup analysis for the association between APTT and septic shock. Subgroup analyses are stratified by age, BMI, gestational weeks, parity, ICU admission status, and clinical stage (antenatal vs. postpartum).

Subgroup analysis stratified by maternal characteristics (Figure 4) assessed the association between APTT and septic shock in pregnant women. The effect size of APTT on septic shock risk remained consistent across most subgroups, with non-significant interaction terms for age, gestational weeks, parity, ICU admission status, and clinical stage (all *p* > 0.05). However, a significant interaction was observed for BMI (*p* for interaction = 0.017). This disparate effect suggests that coagulation dysfunction reflected by prolonged APTT may disproportionately influence septic shock pathogenesis in leaner pregnant women. The underlying BMI-dependent susceptibility mechanisms warrant further investigation in obstetric critical care.

## 4 Discussion

This retrospective cohort study of 1,631 infected pregnant women revealed a U-shaped relationship between APTT and septic shock risk. Significantly, both APTT < 28 s (adjusted OR 1.5) and >42 s (adjusted OR 3.85) conferred substantially higher shock odds compared to the 28–42 s reference range. Threshold analysis identified an inflection point at 27.564 s, with each 1-s APTT increase beyond this point elevating shock risk by 8.7%. The APTT >42 s group exhibited markedly higher ICU admission rates, fetal mortality, severe coagulopathy, multi-organ dysfunction, and greater intervention needs. These findings establish both abnormally short and prolonged APTT values as critical indicators of clinical deterioration in obstetric sepsis.

This study observed an overall maternal mortality rate of 0.3% and a fetal mortality rate of 11.5%. This maternal mortality rate is lower than those reported in many earlier studies on obstetric sepsis (3), which likely reflects recent advances in the management of maternal critical illness and early recognition. Notably, WHO data show a decline in the proportion of global maternal deaths from sepsis, from 10.7% (2003–09) to 7% (2009–20), reinforcing that advances in management and care are reducing sepsis-related mortality (24, 25). However, the persistently high fetal mortality rate highlights that maternal sepsis remains a critical threat to fetal survival, contrasting with the improvement in maternal outcomes. This disparity underscores the need for clinical strategies focused on fetal protection during maternal septic episodes (26). However, in the group with significantly prolonged APTT (>42 s), the maternal mortality rate sharply increases, and the fetal mortality rate is also significantly higher than that in the baseline group. This highlights the extremely high risk faced by patients with severe coagulation dysfunction and further confirms that maternal severe sepsis accompanied by coagulopathy is closely associated with adverse perinatal outcomes. This demonstrates that although the overall maternal sepsis mortality rate may have decreased due to improvements in diagnosis and treatment, cases complicated by severe coagulation disorders remain a major challenge in clinical management.

In our study, subgroup analysis revealed a significant interaction between BMI and the association of APTT with septic shock (*p* for interaction = 0.017), suggesting that the prognostic implication of coagulopathy may be disproportionately greater in leaner pregnant women. This finding supports the “obesity paradox” observed in critical care settings. A meta-analysis of septic ICU patients revealed that both overweight (aOR 0.83, 95% CI 0.75–0.91) and obese individuals (aOR 0.82, 95% CI 0.67–0.99) exhibited a significant reduction in adjusted mortality risk relative to patients with normal weight. Proposed mechanisms for this phenomenon include higher energy reserves, increased capacity to buffer endotoxins, and modified immune-inflammatory responses (27). Another meta-analysis indicates that increased BMI (overweight or obesity) is associated with reduced mortality risk across general sepsis populations. Conversely, low body weight independently predicts sepsis-related mortality. However, in patients aged ≤50 years, the survival advantage associated with higher BMI did not reach statistical significance (28). Considering that our study population consists of pregnant and postpartum women, who represent a relatively young cohort, this finding provides a plausible explanation for the observed phenomenon: younger individuals with low BMI may lack adequate metabolic reserves and immunological buffering capacity, thereby exhibiting greater vulnerability to the severe coagulopathy and inflammatory state indicated by APTT prolongation. However, caution is required when interpreting the association between BMI and septic shock risk among pregnant women, as BMI possesses inherent limitations in this population. Unlike in the general population, gestational weight gain and increased BMI represent multifactorial physiological changes, reflecting fetal and placental mass, amniotic fluid volume, plasma expansion, and physiological oedema. Consequently, BMI is a suboptimal measure for distinguishing fat mass from non-fat components or characterising body fat distribution. Future studies should integrate direct body composition assessment methods to better clarify the underlying pathophysiological mechanisms.

Among various tools for assessing maternal sepsis and septic shock, the Modified Early Obstetric Warning System (MEOWS) is one of the commonly employed bedside clinical tools. Its implementation is recommended to improve early detection of clinical deterioration (29), and it retains predictive value even in resource-limited settings (30). While our study did not evaluate MEOWS directly, our findings on APTT suggest a complementary role. MEOWS monitors overt physiological changes, whereas an abnormal APTT may provide an earlier, objective laboratory signal of developing sepsis-induced coagulopathy. Thus, APTT does not replace but could enhance MEOWS, with the combination potentially enabling a more timely and specific multi-parameter warning system for maternal sepsis.

Few studies have specifically explored the association between APTT and outcomes in maternal septic shock. Previous studies have indicated that coagulation parameters such as PT/INR exhibit moderate diagnostic utility for septic shock and robust prognostic accuracy regarding 30-day all-cause mortality among individuals with sepsis or septic shock (31). Among standard coagulation tests, Bai et al. identified PT as possessing the greatest potential for forecasting sepsis severity and patient outcomes. Specifically, a PT level exceeding 16.5 s at ICU admission showed a significant association with both progression to septic shock and 28-day mortality in sepsis patients (32). Furthermore, Nicolas Chopin et al. proposed that APTT waveform analysis assists in diagnosing severe sepsis and evaluating prognosis for septic patients admitted to the ICU (33). These studies focus primarily on coagulation in general sepsis/septic shock populations, limiting their applicability to maternal sepsis. Our study specifically examines coagulation changes in maternal sepsis, exploring the association between APTT and septic shock development. The prognostic superiority of APTT over INR/PT in maternal septic shock stems from its ability to provide a timelier signal of incipient DIC. APTT reflects the precipitous consumption of coagulation factors with short half-lives, such as factor V (12–36 h) and fibrinogen, more rapidly than INR/PT, which tracks more stable vitamin K-dependent factors. Crucially, APTT also encapsulates the defining haemostatic pathologies of advanced sepsis, including consumptive coagulopathy and platelet dysfunction, into a single parameter, thus consolidating its clinical prognostic utility.

Our research has some advantages. As a simple, low-cost, and globally accessible test, APTT represents a viable biomarker for risk stratification even in resource-constrained settings where the burden of maternal sepsis is disproportionately high. Future research should prioritize the validation of APTT-based monitoring protocols in diverse, low-resource healthcare systems to assess their impact on improving early detection, guiding timely intervention, and ultimately reducing preventable maternal mortality from sepsis worldwide.

However, several limitations warrant consideration when interpreting our findings. First, the single-centre, retrospective design inherently limits generalisability and carries a considerable risk of selection bias and residual confounding not fully captured or adjusted for. Second, the sample size, particularly the small subgroup with extreme APTT prolongation (>42 s; *n* = 19), undermines statistical power for reliable subgroup analyses and may yield imprecise effect estimates. Third, the absence of serial APTT measurements and granular data on infection source (e.g., bacterial versus viral) precludes definitive inference on the temporal dynamics linking coagulation dysregulation to septic shock evolution or severity. Fourth, due to the retrospective nature of our study and reliance on baseline admission data, we did not perform mixing studies or specific factor inhibitor assays. Therefore, we cannot rule out the possibility that acquired hemophilia, a known pregnancy-associated condition, contributed to the prolonged APTT observed in some patients. This limits our ability to precisely delineate the contribution of sepsis-induced coagulopathy versus other causes of coagulation abnormality. Future prospective studies should incorporate such diagnostic evaluations to clarify the specific mechanisms underlying APTT prolongation in maternal sepsis. Furthermore, due to the highly skewed distribution of ICU length of stay in our cohort, we were unable to robustly analyze its association with APTT. Future studies should prospectively evaluate ICU resource use in relation to coagulation parameters in maternal sepsis. In addition, our study did not include specific testing for autoimmune diseases, such as antiphospholipid syndrome, which are known to prolong APTT independently through mechanisms like lupus anticoagulant activity (34). While we adjusted for a range of clinical and laboratory variables, the potential influence of undiagnosed autoimmune conditions on APTT cannot be entirely ruled out. Future prospective studies should consider incorporating autoimmune screening in patients with unexplained coagulation abnormalities to better delineate the specific contribution of sepsis-induced coagulopathy. These constraints underscore the need for prospective, multicentre studies with larger, more diverse cohorts, serial coagulation assays, and detailed microbiological phenotyping before implementing APTT-guided risk stratification in clinical practice.

**Table 3:** Threshold effect analysis of APTT on septic shock using a two-piecewise logistic regression model.

APTT levels	Adjusted model	
	OR (95% CI)	*p*-value
<27.564	0.87 (0.746 ~ 1.013)	0.073
≥27.564	1.087 (1.033 ~ 1.145)	0.0015
Likelihood ratio test		0.009

## 5 Conclusion

This study reveals a critical, non-linear relationship between APTT and septic shock risk in pregnant women with sepsis. We identified a key threshold at 27.564 s; not only does each 1-s increase beyond this point elevate shock risk, but values below it are also independently associated with increased odds of shock. This U-shaped association establishes APTT as a pivotal biomarker, underscoring that both shortening and prolongation serve as warning signs. Integrating APTT monitoring into obstetric sepsis care enables comprehensive risk stratification, urging vigilance across the coagulation spectrum to guide timely intervention and improve outcomes.

## Data Availability

The raw data supporting the conclusions of this article will be made available by the authors, without undue reservation.

## Glossary

ICU: Intensive Care Unit
SIRS: systemic inflammatory response syndrome
DIC: disseminated intravascular coagulation
SIC: Sepsis-induced coagulopathy
IL-6: interleukin-6
TNF-*α*: tumor necrosis factor-alpha
APTT: activated partial thromboplastin time
SOFA: Sequential Organ Failure Assessment
PPH: Postpartum hemorrhage
BMI: body mass index
ECMO: Extracorporeal Membrane Oxygenation
CVVH: continuous veno-venous hemofiltration
GCS: Glasgow Coma Scale
APACHE II: Acute Physiology and Chronic Health Evaluation II
PT: prothrombin time
INR: international normalized ratio
SD: standard deviation
IQR: interquartile range
WBC: White Blood Cell Count
ALT: Alanine Aminotransferase
RCS: restricted cubic splines
MEOWS: Modified Early Obstetric Warning System
